# Prenatal Diagnosis of Ductal Constriction in Normal Heart Anatomy—Are There Any Neonatal Consequences?

**DOI:** 10.3390/jcm14103388

**Published:** 2025-05-13

**Authors:** Łucja Hanna Biały, Tomasz Talar, Ewa Gulczyńska, Iwona Strzelecka, Maria Respondek-Liberska

**Affiliations:** 1 Students’ Prenatal Cardiology Scientific Group, Medical University of Łódź, 92-213 Łódź, Poland; 2 Neonatology Department, Polish Mother’s Memorial Hospital Research Institute, 93-338 Łódź, Poland; 3 Department of Prenatal Cardiology, Polish Mother’s Memorial Hospital Research Institute, 93-338 Łódź, Poland; 4 Department of Fetal Malformations Diagnosis and Prevention, Medical University of Łódź, 92-213 Łódź, Poland

**Keywords:** neonatal hyperbilirubinemia, ductal constriction, normal heart anatomy, fetal echocardiography, prenatal diagnosis

## Abstract

**Background/Objectives**: The main goal of this study was to determine whether ductal constriction in the third trimester of a pregnancy during fetal echocardiography examination has an impact on the neonatal clinical condition during the first days after birth. **Methods:** A retrospective study was based on 348 newborns who were examined during their fetal life in the third trimester of a pregnancy in our fetal cardiology center. They were divided into two groups: the study group (*n* = 49): neonates with “normal heart anatomy” (NHA), assessed by fetal echocardiography (ECHO) examination and prenatally diagnosed ductal constriction (NHA-DC); and the control group (*n* = 299): NHA neonates without DC (NHA-NDC). **Results**: Prenatally, DC was associated with other functional abnormalities, such as myocardial hypertrophy, cardiomegaly, tricuspid regurgitation, pericardial effusion and abnormal flow through foramen ovale. Neonates with prenatally diagnosed DC in 43% of cases presented with elevated neonatal bilirubin levels requiring phototherapy treatment (*p* < 0.006). In the study group 27% of neonates showed signs of breathing difficulties in the first hours of life (*p* < 0.001). Neonates with a prenatal diagnosis of DC were hospitalized longer than neonates with a normal heart study (NHS) (*p* < 0.001). **Conclusions**: Neonates with a prenatal diagnosis of ductal constriction are prone to having transient respiratory problems (up to 27%) and mild neonatal hyperbilirubinemia (in presented series up to 43%). Gestational diabetes can be associated with ductal constriction.

## 1. Introduction

Ductal constriction (DC) is a functional abnormality mostly seen in the third trimester of pregnancy, which can be prenatally detected during fetal echocardiography (ECHO) examination, usually as a change in shape of a ductus arteriosus (DA), an aliasing in Color Doppler, an increased peak systolic velocity (PSV) or a decreased pulsatility index (PI). The fetus itself is most commonly cardiovascularly stable; however, if the DA closes before birth it can lead to serious complications, including fetal demise. After birth, it was proven that it can lead to persistent pulmonary hypertension of the newborn (PPHN) in 18–28% of cases of DC [1].

We aimed to check the importance of a ductal constriction and its impact on a neonatal outcome, especially in a normal heart anatomy fetus.

## 2. Materials and Methods

A retrospective study was made based on selected 348 newborns, out of 6020 examined in our center, with a prenatal diagnosis of “normal heart anatomy” (NHA) and fetal echocardiography examination carried out in their third trimester of pregnancy in our fetal cardiology tertiary center. All neonates with congenital heart defects (CHDs) and extracardiac malformations (ECMs) were excluded from this study. All premature neonates, born before the 37th week of pregnancy, were also excluded from this study. Only singleton pregnancies were included.

**Figure 1:**
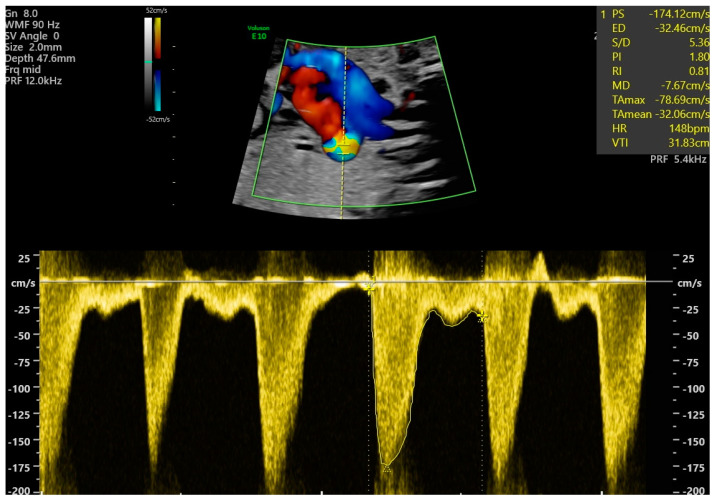
Fetal ductal constriction with a maximal velocity of 174.12 cm/s and PI 1.8, with visible aliasing in ductus arteriosus (DA), both suggesting ductal constriction, at 31 weeks of gestation.

The study group NHA-DC (*n* = 49) was defined as newborns with a prenatal diagnosis of NHA and ductal constriction (DC) in the third trimester of pregnancy (with the minimum gestational age being 28 + 0 and maximum of 39 + 0 weeks of gestation) during fetal echocardiography examination and the control group NHA-NDC (*n* = 299) was planned as newborns prenatally diagnosed as without ductal constriction and with a normal heart study (NHS). NHS was defined by our center as normal heart anatomy (NHA) and possibly only a single functional anomaly, such as tricuspid regurgitation (TR), pericardial effusion (PE) or myocardial hypertrophy. Cases with two or more functional abnormalities were excluded from the NHA-NDC group. All diagnoses were evaluated and confirmed by one investigator. Both groups were analyzed based on maternal factors, functional abnormalities seen in those fetuses in the third trimester of pregnancy and neonatal outcome. Maternal factors were defined as a pregestational diabetes, gestational diabetes, hypertension or maternal obesity with a BMI greater than 30 (Table 1). Fetal functional abnormalities were described as:

- Ductal constriction defined as peak systolic velocity (PSV) > 140 cm/s or > 95th percentiles for a gestational age and diastolic Doppler velocity > 35 cm/s or > 95th percentiles for gestational age, or a decrease in pulsatility index (PI) < 1.9 [1] (Figure 1).
- Cardiomegaly, with a heart area/chest area (HA/CA) > 0.42 [2,3].
- Myocardial hypertrophy with septum thickness > 4.5 mm regardless of gestational age measured in M-Mode view [4].
- Tricuspid regurgitation seen in a four-chamber view (4CV) in a Color Doppler and in Pulsed Wave Doppler with peak systolic velocity > 1.5 m/s and duration > 80 ms [5].
- Bidirectional flow in foramen ovale (right-left and left-right flow) [6].
- Pericardial effusion seen in 4CV as >3 mm of fluid [6].
- Reversal flow in the aortic arch seen in a sagittal view [6].
- Pulmonary valve insufficiency at the level of right ventricular outflow tract (RVOT) or pulmonary trunk seen in Color and Pulsewave Doppler during diastole [6].

In newborns, we assessed gestational age, mode of delivery, the neonate’s birthweight and sex, occurrence of elevated bilirubin level requiring phototherapy and neonatal breathing difficulties, which was described as neonates’ low blood saturation (<90%), changes in X-ray imaging or symptoms of neonate’s respiratory distress, described as increased respiratory effort, increased demand for oxygen or the use of respiratory support. Screening neonatal echocardiography did not reveal any false positive results.

The ultrasound machines Voluson E10 and Samsung Hera W10 Elite with 3–6 MHz probes were used to assess fetal anatomy to exclude any CHDs and ECMs and to perform fetal echocardiography examination. Before the data were introduced to the database, all examinations and results were reviewed and verified by a co-author.

Numeric variables were expressed as mean (±SD) and discrete outcomes as absolute and relative (%) frequencies. Group comparability was assessed by comparing baseline demographic data and follow-up duration between groups. Normality and hetereoskedasticity of continuous data were assessed with Shapiro–Wilk and Levene’s test, respectively. Continuous outcomes were compared with unpaired Student *t*-test, Welch *t*-test or Mann–Whitney U test according to data distribution. Discrete outcomes were compared with chi-squared or Fisher’s exact test accordingly. The alpha risk was set to 5% and two-tailed tests were used. Statistical analysis was performed with Microsoft Excel 2024, Statistica 13.1 programs and EasyMedStat (version 3.37.1; www.easymedstat.com).

## 3. Results

### 3.1. Fetal Functional Abnormalities

In association with ductal constriction, other functional abnormalities occurred, such as a myocardial hypertrophy (31%), reversal flow in the aortic arch (10%), PE (16%), TR (27%), cardiomegaly (29%), a bilateral flow through foramen ovale (18%) and pulmonary valve insufficiency (2%).

### 3.2. Neonatal Outcome

#### 3.2.1. Mode and Gestational Age of Delivery

Median gestational age of delivery was 39.0 (IQR 1.3) in patients with DC and 39.3 (IQR 1.7) in patients without DC (Wilcoxon–Mann–Whitney test, *p* = 0.006), Table 2. By Fisher’s exact test the proportion of groups delivered via C-section, cephalotractor and vaginal birth were, respectively, 65%, 0.0% and 35% in groups of neonates with fetal DC and 53%, 2% and 45% in groups without DC (*p* > 0.05).

#### 3.2.2. Neonates’ Birthweight, Apgar Score and Sex

For the NHA-DC group the median neonatal birthweight was 3337 g, where in the NHA-NDC group it was 3259 g, *p* > 0.05. In neonates with a prenatal diagnosis of DC, the median Apgar Score at the 5th minute was 10.0 (IQR 1.0) with the mean 9.4 (SD 0.9), compared to patients without DC with the median 10.0 (IQR 0.5) and the mean 9.7 (±0.5), *p* = 0.016. The proportion of groups of males and females were, respectively, 57% and 43% in the study group and 46% and 53% in the control group (OR = 0.64; CI [0.35; 1.18]; *p* > 0.05).

#### 3.2.3. Respiratory Problems

**Figure 2:**
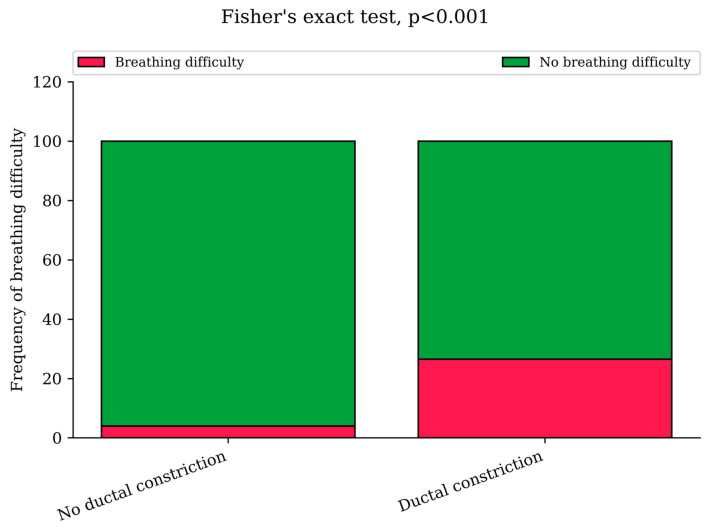
Prevalence of neonatal respiratory problems in the NHA-DC and NHA-NDC groups (Fisher’s exact test, *p* < 0.001), which was described as increased respiratory effort, increased demand for oxygen or use of respiratory support.

In the NHA-DC group, 27% of neonates experienced some form of respiratory problems, compared to the NHA-NDC group with 4% occurrence by Fisher’s test, *p* < 0.001 (Figure 2). Despite their breathing problems, there was no statistical difference in hospitalization times between neonates experiencing respiratory problems in both groups, *p* > 0.05. In both groups it could be seen as a decreased neonatal saturation level requiring oxygen therapy, the X-ray imaging suggesting neonate respiratory distress syndrome (RDS), or as clinical symptoms of respiratory problems in the neonate, such as intercostal retraction, cyanosis or respiratory effort (Table 3). As a breathing difficulty coexisted with diabetes in only two cases (both suffered from GDM type G2 and both were in the group without DC) it did not influence an occurrence of respiratory problems in neonates with a ductal constriction.

#### 3.2.4. Bilirubin

The elevated neonatal bilirubin levels requiring phototherapy rates were, respectively, in the NHA-DC and the NHA-NDC group 43% and 23% (OR = 2.5; CI [1.34; 4.68]; *p* = 0.006). In patients that required phototherapy in the study group (*n* = 21) the mean bilirubin level was 13.18 (±2.01), compared to the control group (*n* = 69) with a mean of 13.32 (±1.74), *p* > 0.05. As a TR is known for causing a mild neonatal hyperbilirubinemia [7], we tested for it in multivariate analysis and for this study group TR was not associated with the rate of an elevated bilirubin level (OR = 0.59, [0.23; 1.47], *p* > 0.05).

#### 3.2.5. Hospitalization Days

The median hospitalization days for the NHA-DC group was 4.0 (IQR 4.0), as for patients in the control group the median was 3.0 (IQR 2.0), *p* < 0.001. Minimal and maximal number of days of hospitalization in the NHA-DC group were 2 and 24 and for the NHA-NDC group it was 2 and 49.

## 4. Discussion

The ductus arteriosus (DA) is a physiological fetal vessel shunt between the main pulmonary artery (MPA) and aortic isthmus. Around 80–85% of low-oxygenated blood goes from right ventricle (RV) to the DA and then to the descending aorta, where it combines with blood coming from the left ventricle (LV) [8]. After birth, the decreased level of prostaglandin E2 (PGE2) means the DA closes [9]. Usually, in full-term healthy neonates it happens between 12 and 24 h after birth. Firstly, by functional closing and then after days or weeks, anatomically [10].

Ductal constriction (DC) can usually be seen in the third trimester of pregnancy and it is often associated with maternal intake of NSAIDs [11,12,13], Indomethacin [14,15] or certain foods and drinks rich in polyphenols (green, black, mate, herbal teas, wine, dark chocolate, orange, red and purple grapes, strawberries and other berries, black prunes, natural juices, olive and soy oils, purple onions, green spices, tomatoes) [8]. Therefore, after fetal echocardiography examination, if we suspect DC in the fetus in the third trimester, in our unit we always warn the patient about effects of those medications and foods connected to DC.

In a fetal echocardiography examination, ductal patency is described via different values, such as peak systolic velocity (PSV), peak diastolic velocity (PDV) and the pulsatility index (PI) [1]. Furthermore, DC may be also associated with other cardiovascular findings, such as myocardial hypertrophy, tricuspid and pulmonary regurgitation, right atrium dilation and cardiomegaly [16]. All those functional cardiovascular anomalies, as expected, were observed in our study. However, in the presented series, those fetuses were also associated with other functional abnormalities, such as abnormal (bidirectional or left-right) flow in the foramen ovale and a reversal flow in the aortic arch, probably due to an increased maximal velocity in the DA. For the differential diagnosis we should also take into account a redundant foramen ovale membrane, which may cause bidirectional foramen ovale blood flow; however, it was not present in this series of cases [17].

Myocardial hypertrophy and fetal cardiomegaly are fetal functional abnormalities that are most commonly associated with maternal diabetes mellitus (DM) [18,19,20]. In the presented series, 35% of women in the study group suffered from DM, whether it was pregestational or gestational diabetes; however, in this series it did not influence the occurrence of myocardial hypertrophy in the study and control groups.

Another observed functional abnormality was the tricuspid regurgitation. In this study, it was observed in 27% of fetuses with DC (*p* < 0.02). As an individual finding, TR is present in approximately 6,8% of singleton fetuses [5].

Fetuses experiencing fetal constriction are mostly hemodynamically stable. To assess that we can use the “Cardiovascular Profile Score” (CVPS), whereby using the fetal ECHO, we assess it using heart size, doppler waveform in the ductus venosus and umbilical vein and umbilical artery and cardiac function (presence of tricuspid or mitral insufficiency) [21].

After birth, 27% of neonates with prenatal DC in our analysis experienced some form of transient respiratory problems. This issue was already analyzed in the literature. Alvarez et al. [1] stated that around 18–28% of cases of DC can lead to a persistent pulmonary hypertension of the newborn (PPHN). In another article by Van Vonderen et al. [22], it was stated that the ductal shunting can influence neonates’ breathing because of an increase in left-to-right shunt due to aspiration of the air. As prenatal DC may precede PPHN of the newborns, in the neonatal screening protocol for neonatal echocardiography the measurement of pulmonary pressure should be added in those cases, or they should be referred for pediatric echocardiography. Moreover, Zielinski et al. [23] asserted that if prenatally the fetal DC is reversed by discontinuing the intake of prostaglandin inhibitors, such as earlier mentioned NSAIDs and food and drink rich in polyphenols, it benefits pulmonary vascular maturation by decreasing the estimated pulmonary artery (PA) pressure.

Usually, after birth physiologic jaundice appears in full-term infants with a peak in the bilirubin level around the 3rd–4th day of life with an average total serum bilirubin (TsB) concentration of 5 mg/dL [24]. However, in some cases its levels can be higher, due to several factors. Hyperbilirubinemia is mostly detected by a visible discoloration of neonates’ skin or mucus membranes. Usually as a mild finding hyperbilirubinemia can be treated with phototherapy, often only ongoing for a couple of days, but if left untreated in some cases it can also lead to serious complications. High levels of bilirubin can lead to damage in the central nervous system causing encephalopathy, deposits in the subcortical nuclei and the spinal cord, leading to disability or in the most severe cases even a neonate’s death [25,26]. However, it is important to state that during the time of this study the modified guidelines for the use of phototherapy came into use from the beginning of 2023 [27].

In the presented series 43% (*p* < 0.006) of neonates with prenatal DC experienced mild neonatal hyperbilirubinemia. In another research study from our center by Respondek-Liberska et al. [7], TR was associated with neonatal mild hyperbilirubinemia in the 4th and 5th day of life. According to the mentioned study, TR could be present during prenatal ECHO examination due to intrauterine infection causing impaired liver function and secondary causing RV functional abnormalities seen as fetal TR. Neonatally, it was seen as mild hyperbilirubinemia with mean 11 mg/dL (range 10–15 mg/dL). In the referred article, up to 46% of neonates with prenatal TR presented with mild hyperbilirubinemia (*p* < 0.0005) [7]. As the number of cases in this series is relatively small, the NHA-DC group was not subdivided to assess the severity of the DC. There was no case of a complete ductal closure. However, for the future it would be important to collect more cases to combine the severity of DC with TR and with elevated bilirubin levels at birth.

Maternal factors of neonatal hyperbilirubinemia include excessive weight gain during pregnancy and complications of pregnancy such as gestational diabetes and hypertension [28,29]. Additionally, it can be caused by many other factors, such as maternal parity and age, as well as a history of jaundice in siblings [30], other factors include hypoxia, acidosis, head hematoma, sepsis and hypoglycemia, as well as infection, hyperbilirubinemia in relation to breastfeeding, alloimmunization or other severe hemolysis [31,32]. Huang et al. in their study also made an observation that in cases of neonates with elevated bilirubin levels, they experienced lower levels of vitamin D, suggesting that neonates with lower levels are prone to hyperbilirubinemia [32,33]. Another reported possible cause of neonatal indirect hyperbilirubinemia is urinary tract infection (UTI) [34], which is why as the authors Baz et al. suggested diagnosing towards UTI as a routine part of examination of the neonate with hyperbilirubinemia [34].

During the study period of our analysis, there was no single fetus with complete DA closure. As we perform analyses of the DC in years 2016–2024, the same period of time taken for analyses of functional TR [7], we may speculate that both DC and TR are the symptoms of the same fetal pathophysiology—a maternal or fetal infection or temporary events during fetal life. As we do not have microbiological or morphological data to support that, they can be signs of a change of hemodynamics during the pregnancy. However, we need more data to support this hypothesis.

## 5. Conclusions

Neonates with a prenatal diagnosis of ductal constriction are prone to having transient respiratory problems (up to 27%) and mild neonatal hyperbilirubinemia (in presented series up to 43%). Gestational diabetes can be associated with ductal constriction.

## Figures and Tables

**Table 1:** Maternal factors in the NHA-DC and the NHA-NDC groups.

	NHA-DC (*n* = 49)	NHA-NDC (*n* = 299)	*p* Value
Maternal age (mean and SD)	30.9 (±5.2)	31.4 (±5.8)	>0.05
Gestational age at exam (mean, SD)	33 (±4.2)	33 (±4.5)	>0.05
Nulliparity (*n*, %)	17 (34.7%)	109 (36.5%)	>0.05
Pregestational diabetes (*n,* %)	3 (6%)	9 (3%)	>0.05
Gestational diabetes (*n*, %)	13 (27%)	39 (13%)	0.016
Hypertension (*n*, %)	15 (30.6%)	17 (5.7%)	<0.001
BMI > 30 (*n*, %)	4 (8%)	30 (10%)	>0.05

**Table 2:** Neonatal outcome in the NHA-DC and the NHA-NDC groups.

	NHA-DC (*n* = 49)	NHA-NDC (*n* = 299)	*p* Value
Gestational age at birth (median, IQR)	39.0 (IQR 1.3)	39.3 (IQR 1.7)	0.006
Birth weight (mean, SD)	3258.9 (SD 450.5)	3271.0 (SD 472.0)	>0.05
5th minute Apgar (median, IQR)	10.0 (IQR 0.5)	10.0 (IQR 1.0)	0.027
5th minute Apgar < 7 (*n*, %)	1 (2%)	2 (0.7%)	>0.05
Neonatal respiratory complications	13 (27%)	12 (4%)	<0.001
Phototherapy for hyperbilirubinemia	21 (43%)	69 (23%)	0.006
Bilirubin level (median, IQR)	13.3 (IQR 2.4)	13.2 (IQR 1.8)	>0.05
Days of hospitalization (median, IQR)	4.0 (IQR 4.0)	3.0 (IQR 2.0)	<0.001

**Table 3:** Breathing difficulty in neonates with and without ductal constriction.

	NHA-DC (*n* = 49)	NHA-NDC (*n* = 299)	*p* Value
Incidence of neonatal breathing difficulties	13 (27%)	12 (4%)	<0.001
Decreased O_2_ saturation treated with oxygen	5 (10.2%)	2 (0.7%)	<0.001
X-ray suggesting RDS	4 (8.2%)	5 (1.7%)	0.008
Clinical signs of respiratory problems (intercostal retraction, cyanosis, respiratory effort)	4 (8.2%)	5 (1.7%)	0.008

## Data Availability

Data are available after reasonable request. The original contributions presented in this study are included in the article/Supplementary Material. Further inquiries can be directed to the corresponding authors.

## Supplementary Materials

The following supporting information can be downloaded at: https://www.mdpi.com/article/10.3390/jcm14103388/s1, Table S1. Occurrence of PGDM and GDM and hyperbilirubinemia in the NHA-DC and NHA-NDC groups. There was a statistical difference in the NHA-DC and NHA-NDC groups in the occurrence of gestational diabetes (*p* = 0.016); however, in those cases there was no statistical difference in the presence of elevated bilirubin levels (*p* > 0.05).

## Abbreviations

The following abbreviations are used in this manuscript:

NHA	Normal heart anatomy
ECHO	Echocardiography
DC	Ductal constriction
NHS	Normal heart study
DA	Ductus arteriosus
PSV	Peak systolic velocity
PI	Pulsility index
PPHN	Pulmonary hypertension of the newborn
CHD	Congenital heart defect
ECM	Extracardiac malformation
TR	Tricuspid regurgitation
PE	Pericardial effusion
HA/CA	Heart area/chest area
4CV	Four-chamber view
RVOT	Right ventricular outflow tract
RDS	Respiratory distress syndrome
MPA	Main pulmonary artery
RV	Right ventricle
LV	Left ventricle
PGE2	Prostaglandin E2
PDV	Peak diastolic velocity
DM	Diabetes mellitus
CVPS	Cardiovascular Profile Score
PA	Pulmonary artery
TsB	Total serum bilirubin
UTI	Urinary tract infection

## References

[1] Alvarez S.G.V., McBrien A. (2018). Ductus arteriosus and fetal echocardiography: Implications for practice. Semin. Fetal Neonatal Med..

[2] Sylwestrzak O., Respondek-Liberska M. (2018). Echocardiographic Methods of Fetal Heart Size Assessmentheart to Chest Area Ratio and Transversal Heart Diameter. Prenat. Cardiol..

[3] Respondek M., Respondek A., Huhta J.C., Wilczynski J. (1992). 2D echocardiographic assessment of the fetal heart size in the 2nd and 3rd trimester of uncomplicated pregnancy. Eur. J. Obstet. Gynecol. Reprod. Biol..

[4] Szmyd B., Biedrzycka M., Karuga F.F., Rogut M., Strzelecka I., Respondek-Liberska M. (2021). Interventricular Septal Thickness as a Diagnostic Marker of Fetal Macrosomia. J. Clin. Med..

[5] Respondek M.L., Kammermeier M., Ludomirsky A., Weil S.R., Huhta J.C. (1994). The prevalence and clinical significance of fetal tricuspid valve regurgitation with normal heart anatomy. Am. J. Obstet. Gynecol..

[6] Respondek-Liberska M. (2019). Diagnostyka Prenatalna USG.

[7] Respondek-Liberska M., Sylwestrzak O., Murlewska J., Biały Ł., Krekora M., Tadros-Zins M., Gulczyńska E., Strzelecka I. (2023). Fetal Third-Trimester Functional Cardiovascular Abnormalities and Neonatal Elevated Bilirubin Level. J. Clin. Med..

[8] Zielinsky P. (2014). Constriction of fetal ductus arteriosus and maternal intake of polyphenol-rich foods. Pregnancy Cardio.

[9] Hung Y.C., Yeh J.L., Hsu J.H. (2018). Molecular Mechanisms for Regulating Postnatal Ductus Arteriosus Closure. Int. J. Mol. Sci..

[10] Coceani F., Baragatti B. (2012). Mechanisms for Ductus Arteriosus Closure. Semin. Perinatol..

[11] Schiessl B., Schneider K.T., Zimmermann A., Kainer F., Friese K., Oberhoffer R. (2005). Prenatal Constriction of the Fetal Ductus Arteriosus—Related to Maternal Pain Medication?. Z. Geburtshilfe Neonatol..

[12] Auer M., Brezinka C., Eller P., Luze K., Schweigmann U., Schwärzler P. (2004). Prenatal diagnosis of intrauterine premature closure of the ductus arteriosus following maternal diclofenac application. Ultrasound Obs. Gyne.

[13] Paladini D., Marasini M., Volpe P. (2005). Severe ductal constriction in the third-trimester fetus following maternal self-medication with nimesulide. Ultrasound Obs. Gyne.

[14] Respondek M., Weil S.R., Huhta J.C. (1995). Fetal echocardiography during indomethacin treatment. Ultrasound Obs. Gyne.

[15] Norton M.E. (1997). Teratogen update: Fetal effects of indomethacin administration during pregnancy. Teratology.

[16] Pugnaloni F., Doni D., Lucente M., Fiocchi S., Capolupo I. (2024). Ductus Arteriosus in Fetal and Perinatal Life. J. Cardiovasc. Dev. Dis..

[17] Uzun O., Babaoglu K., Ayhan Y.I., Moselhi M., Rushworth F., Morris S., Beattie B., Wiener J., Lewis M.J. (2014). Diagnostic ultrasound features and outcome of restrictive foramen ovale in fetuses with structurally normal hearts. Pediatr. Cardiol..

[18] Wilczynski J., Respondek M., Pertynski T. (1993). Fetal Echocardiography (2D and M-mode) in Pregnant Women with Insulin Dependent Diabetes in the Second Half of Pregnancy. Int. J. Prenat. Perinat. Psychol. Med..

[19] Depla A.L., De Wit L., Steenhuis T.J., Slieker M.G., Voormolen D.N., Scheffer P.G., De Heus R., Van Rijn B.B., Bekker M.N. (2021). Effect of maternal diabetes on fetal heart function on echocardiography: Systematic review and meta-analysis. Ultrasound Obs. Gyne.

[20] Suda-Całus M., Dąbrowska K., Gulczyńska E. (2024). Infant of a diabetic mother: Clinical presentation, diagnosis and treatment. Pediatr. Endocrinol. Diabetes Metab..

[21] Huhta J.C. (2004). Guidelines for the Evaluation of Heart Failure in the Fetus With or Without Hydrops. Pediatr. Cardiol..

[22] Van Vonderen J.J., Roest A.A.W., Klumper F.J.C., Hooper S.B., Te Pas A.B. (2017). The effect of breathing on ductus arteriosus blood flow directly after birth. Eur. J. Pediatr..

[23] Zielinsky P., MagalhÃes G.A., Zurita-Peralta J., Sosa-OlavarrÍa A., Marinho G., Van Der Sand L., Sulis N.M., Nicoloso L.H., Piccoli A., Vian I. (2021). Improvement in fetal pulmonary hypertension and maturity after reversal of ductal constriction: Prospective cohort study. Ultrasound Obs. Gyne.

[24] Kaplan M., Maisels M.J. (2021). Natural history of early neonatal bilirubinemia: A global perspective. J. Perinatol..

[25] Olusanya B.O., Kaplan M., Hansen T.W.R. (2018). Neonatal hyperbilirubinaemia: A global perspective. Lancet Child Adolesc. Health.

[26] Chastain A.P., Geary A.L., Bogenschutz K.M. (2024). Managing neonatal hyperbilirubinemia: An updated guideline. JAAPA.

[27] Kemper A.R., Newman T.B., Slaughter J.L., Maisels M.J., Watchko J.F., Downs S.M., Grout R.W., Bundy D.G., Stark A.R., Bogen D.L. (2022). Clinical Practice Guideline Revision: Management of Hyperbilirubinemia in the Newborn Infant 35 or More Weeks of Gestation. Pediatrics.

[28] Domosud J., Kulik-Rechberger B. (2022). Maternal pre-pregnancy BMI and gestational weight gain as risk factors of jaundice in healthy newborns ≥ 37 weeks of gestation. J. Health Inequal.

[29] Özdek S., Kul M., Barış Akcan A., Çekmez F., Aydemir G., Aydınöz S., Karademir F., Süleymanoğlu S. (2016). The effect of the pre-pregnancy weight of the mother and the gestational weight gain on the bilirubin level of term newborn. J. Matern. Fetal Neonatal Med..

[30] Itova T.D., Georgieva V.A. (2022). Prenatal factors for neonatal jaundice. J. IMAB.

[31] Mitra S., Rennie J. (2017). Neonatal jaundice: Aetiology, diagnosis and treatment. Br. J. Hosp. Med..

[32] Huang J., Zhao Q., Li J., Meng J., Li S., Yan W., Wang J., Ren C. (2021). Correlation between neonatal hyperbilirubinemia and vitamin D levels: A meta-analysis. PLoS ONE.

[33] Zhou W., Wang P., Bai Y., Zhang Y., Shu J., Liu Y. (2023). Vitamin D metabolic pathway genes polymorphisms and vitamin D levels in association with neonatal hyperbilirubinemia in China: A single-center retrospective cohort study. BMC Pediatr..

[34] Baz A.M.K., El-Agamy O.A.E.F., Ibrahim A.M. (2021). Incidence of urinary tract infection in neonates with significant indirect Hyperbilirubinemia of unknown etiology: Case-control study. Ital. J. Pediatr..

