# Supplementary material for: Prenatal Diagnosis of Ductal Constriction in Normal Heart Anatomy—Are There Any Neonatal Consequences?
Source: J Clin Med. 2025 May 13;14(10):3388. doi: 10.3390/jcm14103388 (PMC12112037; doi:10.3390/jcm14103388)
Supplement: Supplementary file 1 [file jcm-14-03388-s001.zip › jcm-3551921-supplementary.pdf]

Journal of
Clinical Medicine

MDPI

**Table S1.** Occurrence of PGDM and GDM and hyperbilirubinemia in the NHA-DC and NHA-NDC groups. There was a statistical difference in the NHA-DC and NHA-NDC groups in the occurrence of gestational diabetes ($p = 0.016$); however, in those cases there was no statistical difference in the presence of elevated bilirubin levels ($p > 0.05$).

| | **NHA-DC (*n* = 49)** | **NHA-NDC (*n* = 299)** | ***p* Value** |
|---|---|---|---|
| PGDM | 6% | 3% | >0.05 |
| PGDM + hyperbilirubinemia | 33% (n = 3) | 70% (n = 10) | >0.05 |
| GDM | 27% | 13% | 0.016 |
| GDM + hyperbilirubinemia | 31% (n = 13) | 13% (n = 39) | >0.05 |

 https://doi.org/10.3390/jcm14103388
